# Clinical Outcomes of Pencil Beam Scanning Proton Therapy in Locally Advanced Non-Small Cell Lung Cancer: Propensity Score Analysis

**DOI:** 10.3390/cancers13143497

**Published:** 2021-07-13

**Authors:** Nalee Kim, Jae Myoung Noh, Woojin Lee, Byoungsuk Park, Hongryull Pyo

**Affiliations:** Department of Radiation Oncology, Samsung Medical Center, Sungkyunkwan University School of Medicine, Seoul 06351, Korea; nalee.kim@samsung.com (N.K.); jm2.noh@samsung.com (J.M.N.); wj0616.lee@samsung.com (W.L.); bs1002.park@samsung.com (B.P.)

**Keywords:** proton therapy, pencil beam scanning, intensity-modulated radiotherapy, non-small cell lung cancer, radiation therapy, chemoradiation

## Abstract

**Simple Summary:**

We analyzed the oncologic outcomes and toxicities after intensity-modulated radiation therapy (IMRT) or pencil beam scanning proton therapy (PBSPT) in patients with locally advanced non-small cell lung cancer treated with concurrent chemoradiation therapy. Due to an imbalance in baseline characteristics between IMRT and PBSPT, we used propensity score-based statistical analysis. Regarding radiation therapy planning, PBSPT exhibited superior sparing of the lung, heart, and spinal cord compared to intensity-modulated (photon) radiotherapy in patients with advanced NSCLC. However, PBSPT resulted in higher incidence of grade 3 or more dermatitis and esophagitis compared to IMRT. Despite declined baseline lung function, PBSPT demonstrated a comparable rate of symptomatic radiation pneumonitis compared to IMRT. PBSPT could be an effective and safe treatment technique with comparable locoregional control.

**Abstract:**

This study compared the efficacy and safety of pencil beam scanning proton therapy (PBSPT) versus intensity-modulated (photon) radiotherapy (IMRT) in patients with stage III non-small cell lung cancer (NSCLC). We retrospectively reviewed 219 patients with stage III NSCLC who received definitive concurrent chemoradiotherapy between November 2016 and December 2018. Twenty-five patients (11.4%) underwent PBSPT (23 with single-field optimization) and 194 patients (88.6%) underwent IMRT. Rates of locoregional control (LRC), overall survival, and acute/late toxicities were compared between the groups using propensity score-adjusted analyses. Patients treated with PBSPT were older (median: 67 vs. 62 years) and had worse pulmonary function at baseline (both FEV1 and DLCO) compared to those treated with IMRT. With comparable target coverage, PBSPT exhibited superior sparing of the lung, heart, and spinal cord to radiation exposure compared to IMRT. At a median follow-up of 21.7 (interquartile range: 16.8–26.8) months, the 2-year LRC rates were 72.1% and 84.1% in the IMRT and PBSPT groups, respectively (*p* = 0.287). The rates of grade ≥ 3 esophagitis were 8.2% and 20.0% after IMRT and PBSPT (*p* = 0.073), respectively, while corresponding rates of grade ≥ 2 radiation pneumonitis were 28.9% and 16.0%, respectively (*p* = 0.263). PBSPT appears to be an effective and safe treatment technique even for patients with poor lung function, and it does not jeopardize LRC.

## 1. Introduction

The mainstay treatment for locally advanced non-small cell lung cancer (NSCLC) is concurrent chemoradiation therapy (CCRT), with a median survival of 29 months [1]. Higher radiation therapy (RT) dose to improve locoregional control (LRC) [2] is often limited given the close proximity to the target volume of critical normal organs. 

Besides local control, radiation-related toxicities during RT could reduce patients’ quality of life and treatment compliance, and some toxicities may be lethal [3]. Beginning typically at the second or third week of CCRT, acute RT-induced esophagitis occurred in 4–18% of patients, interfering with appropriate nutritional support and inducing long-lasting dysphagia [4,5]. After several months, some patients experienced symptomatic radiation pneumonitis (RP), which may affect both quality of life and survival [6]. Owing to promising results from systemic treatments, concerns regarding late-onset toxicity (i.e., cardiotoxicity) have also recently increased [6,7]. Therefore, meticulous RT planning is needed to maximize the therapeutic ratio.

Given the physical advantages over photon RT, proton beam therapy (PBT) is expected to increase the therapeutic ratio by delivering a higher dose to the tumor and sparing normal organs. Along with early dosimetric studies [8,9], both retrospective and prospective clinical studies have reported promising results regarding efficacy and safety [10,11,12,13,14]. However, a recent randomized trial comparing PBT with passive scattering to intensity-modulated radiation therapy (IMRT) demonstrated that PBT did not confer a clinical benefit of RP or LRC over IMRT [15]. Nevertheless, technical advances in proton planning for intensity modulation as well as a change in the delivery form from wobbling to scanning are expected to provide further improvement in normal organ sparing and ultimate dose escalation of tumor [16]. Although several early reports of pencil beam scanning proton therapy (PBSPT) showed promising results, these were mainly focused on planning results. There is no randomized trial comparing PBSPT and IMRT in locally advanced NSCLC [17,18,19,20,21].

Herein, we retrospectively reviewed patients with stage III NSCLC treated with CCRT using PBSPT and compared them with those treated with CCRT using IMRT in terms of planning and clinical outcomes.

## 2. Materials and Methods

### 2.1. Patient Population

After approval from the institutional review board of Samsung Medical Center (No. 2020-01-034), we identified 283 patients with locally advanced NSCLC treated with CCRT between November 2016 and December 2018. Patients were excluded if they underwent PBSPT in combination with IMRT (*n* = 12), did not complete RT (*n* = 13), and follow-up details were missing (*n* = 13). Ultimately, we retrospectively reviewed the medical records of 219 patients; 194 patients were treated with IMRT (IMRT group) and 25 patients were treated with PBSPT (PBSPT group), respectively. Informed consent was waived due to the retrospective nature of this study, and the study was performed in accordance with the provisions of the Declaration of Helsinki and Good Clinical Practice guidelines.

Using the Vmax 22 system (SensorMedics, Yorba Linda, CA, USA), spirometric analysis and diffusing capacity of the lungs for carbon monoxide (DLCO) values were assessed according to the American Thoracic Society/European Respiratory Society criteria. After obtaining absolute values of forced expiratory volume in one second (FEV1) and DLCO, the percentage of the predicted values for FEV1 and DLCO was calculated based on a representative Korean population [22]. Moderately low FEV1 and DLCO were defined as 50% ≤ FEV1 < 70% predicted and 40% ≤ DLCO < 60% predicted; severely low FEV1 and DLCO were defined as FEV1 < 50% and DLCO < 40% predicted [23]. 

### 2.2. Radiation Therapy

Based on all available clinical information, the gross tumor volume (GTV) was delineated in the average-intensity projection images reconstructed from 10 breathing-phase, four-dimensional computed tomography (CT) scans. All patients underwent 18F-fluorodeoxyglucose positron emission tomography/computed tomography for determining GTV and detecting distant metastasis at the time of diagnosis. Internal target volume was established by expanding the GTV to include GTV for each phase of the breathing cycle. The clinical target volume (CTV) was generated by extending a 5 mm margin from the GTV. We routinely did not perform elective node irradiation in the uninvolved lymph node region. For planning target volume (PTV), a uniform 5 mm margin was placed on the CTV to account for setup uncertainty. Median total dose of 66 Gy (range, 59.4–74.0) with a fractional dose of 2.2 Gy (range, 2–2.2) was prescribed for PTV. Specifically, 66 Gy in 30 fractions was the most frequently adopted dose schedule in 152 patients (69.4%) followed by 66 Gy in 33 fractions (*n* = 34, 15.5%), 70 Gy in 35 fractions (*n* = 13, 5.9%), 60 Gy in 30 fractions (*n* = 9, 4.1%), 70.4 Gy in 32 fractions (*n* = 4, 1.8%), 70 Gy in 35 fractions (*n* = 4, 1.8%), and 74 Gy in 37 fractions (*n* = 3, 1.4%). For all patients, 97% of the prescribed dose should encompass at least 95% of the CTV. The planning requirements for organ-at-risk were as follows: both lungs V_5GyE_ < 65% (where V_XXGyE_ is defined as the percentage of the volume receiving more than XX GyE), V_10GyE_ < 45%, V_20GyE_ < 35%, mean lung dose < 20 GyE, heart V_40GyE_ < 50%, esophagus maximum dose (D_max_) < 66 GyE, V_45GyE_ < 50%, and spinal cord D_max_ < 45 GyE. 

Treatment planning for IMRT and PBSPT were generated on the Pinnacle treatment planning system, version 9.2 (Royal Phillips Electronics, Miami, FL, USA), and the RayStation (RaySearch Laboratories, Stockholm, Sweden), respectively. For IMRT, volumetric arc-modulated therapy was mostly adopted (*n* = 119, 61.3%), followed by step-and-shoot method with 6 median coplanar beams (range 6–10) of 6 MV photons (*n* = 71, 36.6%), and Tomotherapy (*n* = 4, 2.1%, Hi-Art TomoTherapy; Accuray, Madison, WI, USA). Regarding volumetric arc-modulated therapy, partial arc angle was used to minimize the radiation exposure of normal lung: 20–280° (±10)/240–180° (±10) and 340–80° (±10)/120–180° (±10) for right and left lung cancer, respectively. 

The relative biological effectiveness (RBE) for PBSPT was considered as a fixed value of 1.1. Most PBSPT plans were calculated under pencil beam algorithm (*n* = 21, 84.0%) followed by Monte Carlo algorithm (*n* = 4, 16.0%). Pencil beam algorithm, considering patients as a stack of semi-infinite layers, models the treatment beam with a summation of narrow pencil beam which interacts with medium for delivering energy [16]. In addition, a single-field optimization (*n* = 23, 92.0%) with 2 fields (*n* = 18, 72.0%) rather than 3 fields was utilized. All fields were delivered in the same day. For all patients in the PBSPT group, the continuous line-scanning method was used; detailed information on beam delivery and treatment procedure have been described previously [24]. Briefly, all PBSPT plans were robustness-optimized plans using minimax optimization [25]. Setup and range uncertainty was addressed as 5 mm and +/−3.5%, respectively. 

Daily image guidance was performed with kilovoltage or megavoltage cone beam CT for IMRT and orthogonal kilovoltage X-ray images/or cone beam CT provided by VeriSuite (MedCom, Darmstadt, Germany) before each treatment session. 

For additional dosimetric comparisons, matched IMRT plans were generated for the corresponding 25 patients in the PBSPT group. Matched IMRT plans were calculated with volumetric arc-modulated therapy and generated under the condition of achieving acceptable target coverage.

### 2.3. Chemotherapy

Overall, 206 patients (94.1%) were treated with the paclitaxel/cisplatin regimen, 8 with paclitaxel/carboplatin, 4 with etoposide/cisplatin, and 1 with cisplatin alone. The paclitaxel/cisplatin or carboplatin regimen consisted of 6 cycles of weekly intravenous paclitaxel (50 mg/m^2^) with cisplatin (25 mg/m^2^) or carboplatin (area under curve of 1.5). The first dose of chemotherapy was delivered on the first day of RT, and additional consolidation chemotherapy was performed following CCRT. There were 20 patients without epidermal growth factor receptor mutation who received consolidative durvalumab (monoclonal PD-L1 antibody) after CCRT: 1 and 19 patients in the PBSPT and IMRT group, respectively. 

### 2.4. Surveillance

Once the planned treatment was completed, patients underwent chest CT, pulmonary function test (PFT), and/or positron emission tomography/CT scan at 1 month after the planned CCRT, as well as every 3 months for the first 3 years and every 6 months thereafter. Local failure was defined as recurrence within the PTV; recurrent regional nodes outside the PTV were considered as regional failures. Recurrences beyond the primary and regional sites were denoted as distant failures. The acute and late toxicity events noted during and after RT were assessed by the treating physicians based on the Common Terminology Criteria for Adverse Events (CTCAE, ver 5.00). Absolute changes in PFT were calculated based on pre-treatment PFT values for available patients. Major cardiac adverse events were defined based on AHA/ACC guidelines: cardiac death, acute myocardial infarction, unstable angina hospitalization, and heart failure [26].

### 2.5. Statistical Analysis

Differences in continuous variables between the two groups were analyzed with Student’s t-test (normally distributed data) and Mann–Whitney U test (non-normally distributed data). The Chi-square test or Fisher’s exact test was used to evaluate differences in categorical variables between the two groups. The Wilcoxon signed rank test for non-parametric paired data was used to compare the PBSPT and paired IMRT plans. All events (including loco-regional failure and death) were measured from the day of CCRT to the time of the event. The Kaplan–Meier analysis was used to estimate LRC and OS. Multivariable analyses of LRC and OS were performed using Cox regression analysis; logistic regression analysis was used to identify the prognostic factors for grade ≥ 3 esophagitis and grade ≥ 2 RP. Factors with *p* < 0.10 in univariable analysis were further assessed in multivariable analysis. Propensity scores were calculated using a multivariate logistic regression model, including sex (female vs. male), age (continuous), pathology (adenocarcinoma vs. non-adenocarcinoma), T stage (T1–2 vs. T3–4), N stage (N2 vs. N3), predicted value of FEV1 (continuous), predicted value of DLco (continuous), and PTV (continuous). Each patient was then assigned an estimated propensity score based on the patient’s baseline characteristics. First, patients were matched using 1:2 optimal matching with a caliper distance set at 0.05 standard deviations of the logit of the propensity scores. Second, stabilized inverse probability of treatment weighting (IPTW) was used to adjust for any covariable imbalance. The standardized mean difference was used to evaluate the balance of covariate distribution between the 2 groups. A two-tailed *p* < 0.05 was considered statistically significant. All statistical analyses were performed using IBM SPSS Statistics version 25 (IBM Corp., Armonk, NY, USA) and R (version 3.6.3; R Foundation for Statistical Computing, Vienna, Austria).

## 3. Results

### 3.1. Baseline Characteristics

In the studied patient population, the median age of the patients was 62 (interquartile range, 57–68) years, and most patients (97.7%) had a good performance status of ECOG PS 0–1 (Table 1). Patients in the PBSPT group were older (median, 67 vs. 62, *p* = 0.003) and had less frequent contralateral mediastinal lymph node involvement (20.0% vs. 43.3%, *p* = 0.044) than those in the IMRT group. The median FEV1 (percentage predicted) and DLCO (percentage predicted) values in the PBSPT group were significantly lower than those in the IMRT group (both *p* < 0.05, Figure S1). In addition, the prevalence of severely low FEV1 and moderately to severely low DLCO in the PBSPT group (20.0% and 40.0%, respectively) was higher than in the IMRT group (4.6% and 15.5%, respectively). 

### 3.2. Radiation Therapy Characteristics

**Figure 1:**
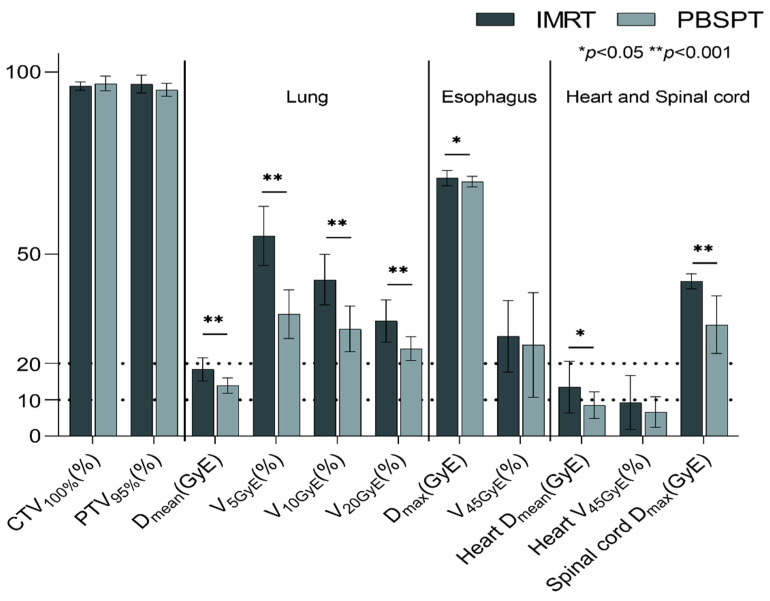
Dose–volume parameters for target volume and normal organs in patients treated with IMRT and PBSPT. Data are presented as the median, interquartile range. *Abbreviations*: IMRT, intensity-modulated radiation therapy; PBSPT, pencil beam scanning proton therapy; GyE, gray equivalent; CTV, clinical target volume; PTV, planning target volume; V_XX%_, volume receiving XX% of the prescription dose; V_XXGyE_, volume receiving more than XX Gy; D_mean_, mean dose; D_max_, maximum dose.

There was no significant difference in total prescription dose and target volumes between the two groups (Figure 1, Table S1). Although PBSPT plans covered 95% of PTV with a lower dose than IMRT plans (94.8% vs. 97.1%, *p* = 0.013), both plans encompassed 100% of CTV under the acceptable institutional criteria, presenting 96.2% and 96.7% of the prescribed dose, respectively (*p* = 0.314). Regarding both lungs, PBSPT significantly reduced not only the average dose but also V_5GyE_, V_10GyE_, and V_20GyE_ (all *p* < 0.001). Although Dmax of esophagus in the IMRT group was higher than that in the PBSPT group (71.2 vs. 69.7 GyE, *p* = 0.042), V_45GyE_, V_55GyE_, and V_66GyE_ were comparable between the two groups (all *p* > 0.05). Plans in the PBSPT group also showed lower mean heart dose (7.7 vs. 12.8 GyE, *p* = 0.006) and D_max_ of spinal cord (31.0 vs. 42.6 GyE, *p* < 0.001) than those in the IMRT group.

### 3.3. Oncologic Outcomes

**Figure 2:**
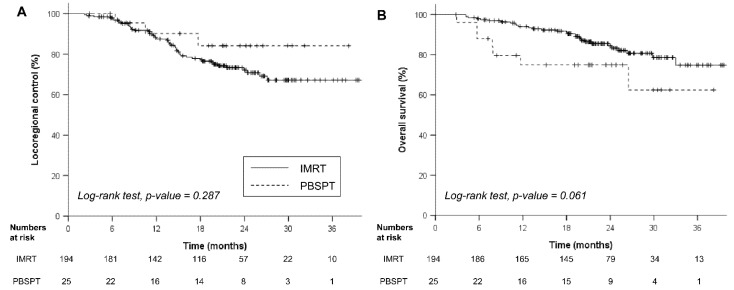
Clinical outcomes after IMRT and PBSPT: LRC (**A**) and OS (**B**). Abbreviations: IMRT, intensity-modulated radiation therapy; PBSPT, pencil beam scanning proton therapy; LRC, locoregional control; OS, overall survival.

With a median follow-up of 21.7 (interquartile range, 16.8–26.8) months for the entire cohort, the rates of 2-year LRC and OS were 72.8% and 82.9%, respectively. During the last follow-up, 50 (22.8%) and 117 patients (53.4%) experienced locoregional failures and distant metastases. The rates of 2-year LRC were 72.1% and 84.1% in the IMRT and PBSPT groups, respectively (*p* = 0.287, Figure 2A). Patients in the PBSPT group showed lower OS rates than those in the IMRT group (rates of 2-year OS: 74.9% vs. 84.4%, *p* = 0.061, Figure 2B). Multivariable analysis revealed that treatment modality had little impact on both LRC and OS; only GTV ≥ 100 cc showed borderline significance in LRC (HR 1.74, *p* = 0.069, Table 2). 

### 3.4. Toxicity

**Figure 3:**
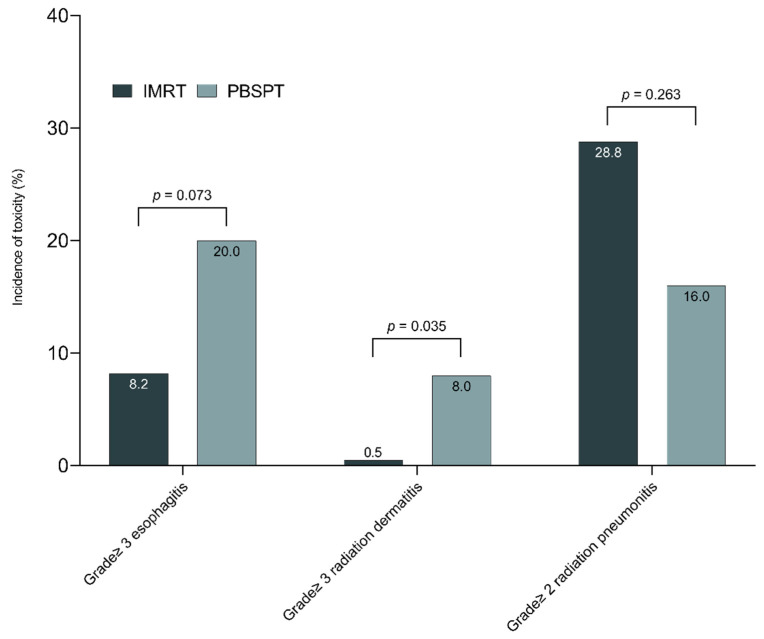
The incidence of specific treatment-related toxicities in patients treated with IMRT and PBSPT. Abbreviations: IMRT, intensity-modulated radiation therapy; PBSPT, pencil beam scanning proton therapy.

The toxicities reported in this study are summarized in Table 3. Twenty-four (11.0%) grade 3 or more acute toxic events were observed in the entire cohort. Among 21 patients with grade 3 or more esophagitis, all patients were hospitalized with temporary total parenteral nutrition and five patients required tube feeding. There was a trend toward frequent grade ≥ 3 esophagitis with PBSPT (20.0% vs. 8.2%, *p* = 0.073, Figure 3); grade ≥ 3 radiation dermatitis was more frequently observed in the PBSPT group than the IMRT group (8.0% vs. 0.5%, *p* = 0.035). PBSPT was associated with frequent grade ≥3 esophagitis after multivariable analysis (odds ratio (OR) 3.68, Table 4). Additionally, esophagus V_45GyE_ ≥ 35% was also related to the incidence of grade ≥ 3 esophagitis in multivariable analysis. There were two patients in the IMRT group who experienced trachea-esophageal fistula requiring surgical intervention. There were 60 patients (27.4%) who experienced symptomatic RP with comparable incidence between the IMRT and PBSPT groups (28.9% vs. 16.0%, *p* = 0.263). Multivariable analysis showed that both-lung V_10GyE_ ≥ 45% significantly increased the grade ≥ 2 RP (OR 4.37, Table 4). Differences in declined pulmonary function between the IMRT and PBSPT groups were not statistically significant throughout the follow-up period (Figure S2). Regarding cardiac adverse events, there was no significant difference between the IMRT and PBSPT groups (9.3% vs. 8.0%, Table 4). 

### 3.5. Dosimetric Comparison for Matched IMRT and PBSPT Plans

After propensity score matching, 50 patients from the IMRT group and 25 patients from the PBSPT group were included following analysis with well-balanced baseline characteristics. In addition, baseline characteristics, except for T stage, were adequately balanced after IPTW (Table 5). 

**Figure 4:**
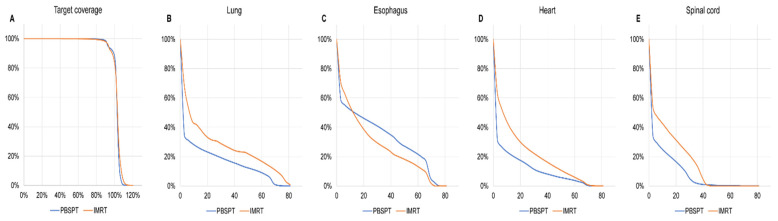
Dose–volume histogram for an average of matched intensity-modulated radiation therapy (IMRT) and pencil beam scanning proton therapy (PBSPT) plans: planning target volume (**A**); both lungs (**B**); esophagus (**C**); heart (**D**); spinal cord (**E**). Abbreviations: IMRT, intensity-modulated radiation therapy; PBSPT, pencil beam scanning proton therapy.

A dose–volume histogram for an average of matched IMRT and PBSPT plans is shown in Figure 4. Although both plans achieved similar CTV/PTV coverage under the institutional dose constraints, PBSPT significantly reduced the volume of lungs, heart, and spinal cord exposed to low-to-high doses of radiation (Table S2). On the contrary, PBSPT increased the intermediate-to-high doses delivered to the esophagus (45, 55, and 66 GyE), whereas PBSPT delivered similar maximum doses to the esophagus (69.7 vs. 71.0 GyE, *p* = 0.241) when compared to matched IMRT plans.

### 3.6. Oncologic and Toxicity Outcomes for Propensity Score-Adjusted Patients

**Figure 5:**
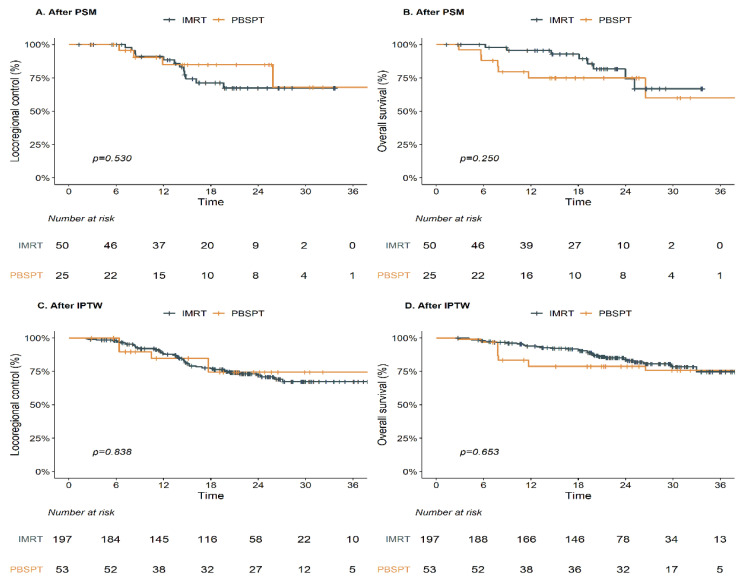
Clinical outcomes following IMRT and PBSPT after propensity score-adjusted analysis: LRC after PSM (**A**); OS after PSM (**B**); LRC after IPTW (**C**); OS after IPTW (**D**). Abbreviations: IMRT, intensity-modulated radiation therapy; PBSPT, pencil beam scanning proton therapy; LRC, locoregional control; OS, overall survival; PSM, propensity score matching; IPTW, inverse probability of treatment weighting.

After PSM and IPTW, PBSPT showed comparable LRC and OS outcomes (Figure 5, Table 6). Regarding toxicity, PBSPT was associated with frequent grade ≥ 3 esophagitis after IPTW (OR 5.33, Table 6). In addition, PBSPT showed a borderline benefit over IMRT for grade 2 or more RP in propensity score-adjusted analyses (Table 6).

## 4. Discussion

Given the recent advances of the scanning beam in PBT, the current results support the early clinical feasibility of PBSPT in definitive CCRT for NSCLC. PBSPT plans significantly reduced the radiation dose to the lung and spinal cord, with comparable target coverage. Showing comparable survival outcomes, PBSPT resulted in similar rates of symptomatic RP, even in patients who were relatively elderly and in those with poor pulmonary function, compared to those treated with IMRT. However, PBSPT was associated with frequent severe acute esophagitis, even with comparable dosimetric results. 

Several plan comparison studies demonstrated that PBT with passive scattering could reduce the volume of lung, esophagus, and spinal cord by up to 30% compared to three-dimensional conformal RT or even IMRT [8,9]. However, a recent randomized trial on PBT with passive scattering showed no benefit compared to IMRT in the doses to normal lung (mean, 16.1 vs. 16.6 Gy), resulting in no significant difference in grade ≥3 RP (10.5% vs. 6.5%) [15]. A possible reason for these conflicting results might result from the technical issues associated with three-dimensional PBT with passive scattering. Recent planning studies of PBSPT showed significant improvements in sparing normal organs [17,18,19,20]. In the current study, both dosimetric results from the entire cohort and head-to-head plan comparison outcomes show consistent results of sparing dose to the normal lung, spinal cord, and heart. Further technical advancements in scanning performance and calculation algorithms could increase both the robustness and advantage in normal tissue sparing [16]. 

The incidence of grade ≥ 2 (24.0%) or grade ≥ 3 (8.0%) acute skin toxicities after PBSPT in the current study was relatively higher than historical data of <5% regarding severe dermatitis (wet desquamation). A relatively high rate of grade ≥ 3 dermatitis after PBT for treating NSCLC has been reported, ranging from 6% to 24% [10,11,12,14]. Concerns remain regarding the increased dermatitis after PBT due to a higher entry dose of the spread-out Bragg peak of protons or the limited number of beams (2–3 per patient) to minimize the radiation dose to the normal lung [14,27,28]. Regarding dose constraints concerning skin, further cost function in planning PBSPT could reduce potential severe dermatitis [29]. The incidence of 20% for grade ≥ 3 esophagitis seems comparable to that of the 18% obtained in the meta-analysis from a historical randomized trial of photon RT [4], while it appears to be more frequent than that of the 13.2% reported in the IMRT group from the secondary analysis of the RTOG 0617 trial [30]. A recent systemic review identified the dose–volume relationship in esophagitis regarding V60GyE, and the current study demonstrated that V45GyE ≥ 35% is associated with esophagitis [5]. However, further analysis adjusted for dose–volume parameters and IPTW analysis demonstrated that PBSPT could increase severe esophagitis despite the comparable dose distribution. We could speculate several possible reasons for frequent severe esophagitis, including robust optimization methods and RBE. Robust optimizations with additional margins to compensate for dose uncertainty could broaden distal falloff and stiffness of target coverage, resulting in unexpected exposure to the esophagus by the beam angle. Although a fixed value of 1.1 is commonly used as RBE for PBT, RBE itself shows various values according to depth, with the highest value observed near the distal edge of the beam [31]. However, further investigation regarding these technical and biological issues related to toxicities is required. 

Since the incidence of grade ≥ 4 RP after photon RT was relatively high, ranging from 18.2% to 35.7%, in patients with poor lung function [32,33], physicians were forced to compromise CTV/PTV coverage or reduce the total dose to prevent severe lung toxicity in such patients [34]. In the current study, although the PBSPT group had poor baseline pulmonary function, there was no grade ≥ 3 RP in the PBSPT group, and the pattern of changes in PFT was similar to that in the IMRT group. Since both mean lung dose and lung V_5GyE-20GyE_ have been reported to be associated with RP [6], PBSPT might influence the RP development. Despite an absence of long-term follow-up for cardiotoxicity in the current study, PBSPT could potentially reduce the radiation-induced cardiac toxic events resulting from reduced mean dose and V_30GyE-50GyE_ of the heart [6]. Atkins et al. suggested stringent avoidance of cardiac radiation dose based on an increased risk of cardiotoxicity and mortality with increasing cardiac dose in patients with locally advanced NSCLC [7]. The recent post hoc modeling study of the RTOG 0617 trial also showed a relationship between a higher dose to cardiopulmonary substructures and unexpected mortality [35]. The reduced dose to those structures of PBT might translate into improved survival of patients undergoing PBT compared to IMRT, which was observed in the National Cancer Database; this potential benefit could thus be maximized when adopting PBSPT [36]. Although there was no difference in major cardiac events between the PBSPT and IMRT groups in the current study, further follow-up of the current study and a more recent randomized trial would further validate the reduced incidence of cardiotoxicity after PBT and demonstrate a survival benefit. 

There are some potential drawbacks in utilizing PBSPT. First, overall costs of PBT easily exceed those of photon RT, even after toxicity rate-adjusted analysis [37]. However, PBT increased the quality-adjusted life-years by 0.549 and 0.452 compared to 3D CRT/IMRT [38]. An ongoing RTOG 1308 trial (ClinicalTrials.gov: NCT01993810) will address cost effectiveness. Further cost-effectiveness analysis in patients with poor lung function should be considered. Second, although 31 centers are available for PBT in the United States [39], there is limited availability of PBT in other regions due to the higher cost of infrastructures relative to photon RT. Therefore, clear evidence demonstrating obvious clinical benefit is needed to justify the implementation of PBSPT in CCRT for NSCLC. 

Although propensity score-adjusted analysis was undertaken, a major confounder cannot be adjusted due to the small sample size of the PBSPT group. Second, as a retrospective study, the physician-assessed toxicities should be interpreted with caution. However, our analysis was strengthened by use of PBSPT and by our inclusion of patients with poor pulmonary function. A recent randomized trial only included patients with FEV1 > 1.0 L; however, we observed 40% of patients in the PBSPT group with moderate-to-severe impaired pulmonary function. In addition, thorough individualized plan analysis not only for the entire patient cohort but also for the parallel patients in the PBSPT group could provide more detailed information. The relatively lower OS of the PBSPT group than the IMRT group might stem from a difference in age distribution; there was no difference in OS outcomes after PSM and IPTW analysis.

In conclusion, we note a possible benefit of PBSPT regarding tolerable toxicities with comparable survival outcomes based on real-world clinical data. Further randomized trials might be warranted to endorse PBSPT as an alternative treatment option in locally advanced NSCLC.

## Figures and Tables

**Table 1:** Patient, tumor, and treatment characteristics.

Variables		IMRT *n* = 194	PBSPT *n* = 25	SMD	*p*-Value
Sex	Male	150 (77.3)	21 (84.0)	0.170	0.615
	Female	44 (22.7)	4 (16.0)		
Age, years		62.0 [56.0; 67.0]	67.0 [61.0; 75.0]	0.685	0.003
ECOG	0–1	191 (98.5)	23 (92.0)	0.306	0.101
Smoking history	Never smoker	44 (22.7)	2 (8.0)	0.416	0.151
	Current or ex-smoker	150 (77.3)	23 (92.0)		
Histology	Squamous cell carcinoma	75 (38.7)	15 (60.0)	0.468	0.067
	Adenocarcinoma	114 (58.8)	9 (36.0)		
	Etc.	5 (2.6)	1 (4.0)		
	NOS	3	1		
	LCNEC	2			
	EGFR mutant, *n* (%)	37 (19.1)	2 (8.0)	0.328	0.266
	ALK translocation, *n* (%)	6 (3.1)	0 (0.0)	0.253	1.000
Clinical T stage	cT1–2	126 (64.9)	15 (60.0)	0.102	0.791
	cT3–4	68 (35.1)	10 (40.0)		
Clinical N stage	cN2	72 (26.8)	13 (52.0)	0.534	0.018
	cN3	142 (73.2)	12 (48.0)		
N3 involvement region	Contralateral mediastinum	84 (43.3)	5 (20.0)	0.517	0.044
	Supraclavicular	71 (36.6)	9 (36.0)	0.012	1.000
Overall stage	IIIA	23 (11.9)	9 (36.0)	0.604	0.008
	IIIB	138 (71.1)	12 (48.0)		
	IIIC	33 (17.0)	4 (16.0)		
Pre-treatment pulmonary function test					
FEV1, L		2.50 (2.10; 3.06)	2.35 (1.61; 2.81)	0.487	0.040
FEV1, %		84.0 (71.0; 95.0)	72.0 (57.0; 88.0)	0.420	0.042
FEV1 < 70% (moderate to severe)		44 (22.7)	10 (40.0)		0.100
FEV1 < 50% (severe)		9 (4.6)	5 (20.0)		0.013
DLco, %		79.5 (66.0; 92.0)	65.0 (51.0; 79.0)	0.654	0.002
DLco < 60% (moderate to severe)		30 (15.5)	10 (40.0)		0.006
DLco < 40% (severe)		2 (1.0)	1 (4.0)		0.306

Values are presented as number of patients (%) or median (interquartile range). Abbreviations: IMRT, intensity-modulated radiation therapy; PBSPT, pencil beam scanning proton therapy; SMD, standardized mean difference; SD, standard deviation; IQR, interquartile range; NOS, not otherwise specified; LCNEC, large cell neuroendocrine carcinoma; EGFR, epidermal growth factor receptor; ALK, anaplastic lymphoma kinase; FEV1, forced expiratory volume in 1 s; DLco, diffusing capacity of the lung for carbon monoxide.

**Table 2:** Prognostic factors for locoregional control and overall survival.

**Locoregional Control**		**Univariable Analysis**			**Multivariable Analysis**		
**Variables**	**(Reference vs.)**	**HR**	**(95% CI)**	***p*-Value**			
RT modality	(IMRT vs. PBSPT)	0.54	(0.17–1.72)	0.296	0.43	(0.13–1.41)	0.165
Sex	(female vs. male)	1.80	(0.84–3.83)	0.130			
Age	(<65 vs. ≥65 years)	0.68	(0.37–1.25)	0.218			
Histology	(non-ADC vs. ADC)	0.52	(0.30–0.92)	0.024	0.58	(0.33–1.05)	0.071
EGFR mutation	(wildtype vs. mutant)	0.51	(0.22–1.20)	0.122			
Clinical T stage	(T1–2 vs. T3–4)	1.50	(0.85–2.63)	0.162			
Clinical N stage	(N2 vs. N3)	0.94	(0.50–1.78)	0.858			
Contralateral mediastinal lymph node	(no vs. yes)	1.20	(0.69–2.10)	0.515			
SCF lymph node	(no vs. yes)	0.81	(0.45–1.46)	0.486			
GTV	(<100 vs. ≥100 cc)	1.90	(1.06–3.38)	0.030	1.74	(0.96–3.16)	0.069
PTV	(<550 vs. ≥550 cc)	1.18	(0.68–2.05)	0.567			
Fractional RT dose	(2.0 vs. 2.2 GyE)	0.21	(0.01–3.86)	0.294			
Total RT dose	(≤66 vs. >66 GyE)	0.97	(0.38–2.44)	0.944			
BED_10_	(≤80 vs. >80 GyE)	0.75	(0.41–1.38)	0.353			
**Overall survival**		**Univariable**			**Multivariable**		
**Variables**	**(reference vs.)**	**HR**	**(95% CI)**	***p*-value**	**HR**	**(95% CI)**	***p*-value**
RT modality	(IMRT vs. PBSPT)	2.17	(0.95–4.94)	0.066	1.64	(0.70–3.81)	0.254
Sex	(female vs. male)	2.17	(0.95–4.94)	0.294			
Age	(<65 vs. ≥65 years)	1.56	(0.68–3.58)	0.030	1.66	(0.82–3.36)	0.156
Histology	(non-ADC vs. ADC)	2.10	(1.08–4.10)	0.006	0.54	(0.26–1.13)	0.103
EGFR mutation	(wildtype vs. mutant)	0.55	(0.16–1.17)	0.098	0.66	(0.21–2.00)	0.459
Clinical T stage	(T1–2 vs. T3–4)	1.28	(0.66–2.49)	0.470			
Clinical N stage	(N2 vs. N3)	0.65	(0.33–1.31)	0.230			
Contralateral mediastinal lymph node	(no vs. yes)	0.85	(0.44–1.64)	0.625			
SCF lymph node	(no vs. yes)	0.98	(0.50–1.90)	0.950			
FEV1 (%)	(≥70 vs. <70%)	0.85	(0.39–1.86)	0.687			
DLco (%)	(≥60 vs. <60%)	1.64	(0.75–3.61)	0.216			
GTV	(<100 vs. ≥100 cc)	1.48	(0.77–2.86)	0.243			
Total RT dose	(>66 vs. ≤66 GyE)	4.55	(0.63–33.3)	0.132			
BED_10_	(>80 vs. ≤80 GyE)	1.01	(0.48–2.12)	0.986			

The foreparts of the parentheses were set as the reference group. Abbreviations: HR, hazard ratio; CI, confidence interval; RT, radiation therapy; IMRT, intensity-modulated radiation therapy; PBSPT, pencil beam scanning proton therapy; ADC, adenocarcinoma; EGFR, epidermal growth factor receptor; SCF, supraclavicular fossa; GTV, gross tumor volume; PTV, planning target volume; GyE, gray relative biologic effectiveness; BED_10_, biological effective dose with α/β of 10; FEV1, forced expiratory volume in 1 s; DLco, diffusing capacity of the lung for carbon monoxide.

**Table 3:** Detailed profiles of treatment-related adverse events *.

Category	Grade	IMRT *n* = 194 *n* (%)	PBSPT *n* = 25 *n* (%)	*p*-Value
Acute toxicity				
Esophagitis	Grade 0	29 (14.9)	4 (16.0)	0.286
	Grade 1	69 (35.6)	9 (36.0)	
	Grade 2	80 (41.2)	7 (28.0)	
	Grade 3	15 (7.7)	5 (20.0)	
	Grade 4	1 (0.5)	0 (0.0)	
Radiation dermatitis	Grade 0	161 (83.0)	19 (76.0)	0.013
	Grade 1	17 (8.8)	0 (0.0)	
	Grade 2	15 (7.7)	4 (16.0)	
	Grade 3	1 (0.5)	2 (8.0)	
Late toxicity				
Radiation pneumonitis	Grade 0	115 (59.3)	18 (72.0)	0.648
	Grade 1	23 (11.9)	3 (12.0)	
	Grade 2	47 (24.2)	4 (16.0)	
	Grade 3	9 (4.6)	0 (0.0)	
Late esophageal toxicity	Grade 0	191 (98.5)	24 (96.0)	0.386
	Grade 1	0 (0.0)	0 (0.0)	
	Grade 2	1 (0.5)	1 (4.0)	
	Grade 3	2 (1.0)	0 (0.0)	
Major cardiac adverse events		18 (9.3)	2 (8.0)	1.000

* Grades refers to grading systems of Common Terminology Criteria for Adverse Events. Abbreviations: IMRT, intensity-modulated radiation therapy (photon); PBSPT, pencil beam scanning proton therapy.

**Table 4:** Prognostic factors for grade ≥ 3 acute esophagitis and grade ≥ 2 radiation pneumonitis.

**Grade ≥ 3 Acute Esophagitis**		**Univariable Analysis**			**Multivariable Analysis**		
**Variables**	**(Reference vs.)**	**OR**	**(95% CI)**	***p*** **-Value**	**OR**	**(95% CI)**	***p*** **-Value**
RT modality	(IMRT vs. PBSPT)	2.78	(0.84–8.00)	0.070	3.68	(0.97–12.88)	0.045
Sex	(female vs. male)	0.33	(0.13–0.85)	0.019	0.32	(0.06–1.74)	0.181
Age	(<65 vs. ≥65 years)	1.2	(0.47–2.98)	0.698			
Smoking history	(never vs. ever)	0.39	(0.15–1.04)	0.049	0.74	(0.13–4.30)	0.735
Clinical T stage	(T1–2 vs. T3–4)	0.89	(0.33–2.26)	0.818			
Clinical N stage	(N2 vs. N3)	0.53	(0.21–1.35)	0.170			
Contralateral mediastinal lymph node	(no vs. yes)	0.89	(0.34–2.21)	0.803			
SCF lymph node	(no vs. yes)	0.86	(0.31–2.16)	0.749			
PTV	(<550 vs. ≥550 cc)	1.39	(0.56–3.54)	0.479			
Fractional RT dose	(2.0 vs. 2.2 GyE)	2.37	(0.76–10.38)	0.181			
Total RT dose	(≤66 vs. >66 GyE)	0.69	(0.27–1.66)	0.408			
Esophagus D_max_	(<70 vs. ≥70 GyE)	2.65	(0.94–9.46)	0.090	2.46	(0.68–10.66)	0.193
Esophagus V_45GyE_	(<35 vs. ≥35%)	4.33	(1.73–11.50)	0.002	3.98	(1.12–19.02)	0.049
Esophagus V_55GyE_	(<20 vs. ≥20%)	3.14	(1.18–9.89)	0.032	0.76	(0.13–3.69)	0.736
Esophagus V_66GyE_	(<10 vs. ≥10%)	2.05	(0.83–5.24)	0.122			
**Grade** **≥ 2 radiation pneumonitis**		**Univariable analysis**			**Multivariable analysis**		
**Variables**	**(reference vs.)**	**OR**	**(95% CI)**	***p*** **-value**	**OR**	**(95% CI)**	***p*** **-value**
RT modality	(IMRT vs. PBSPT)	0.47	(0.13–1.30)	0.183	0.88	(0.24–2.64)	0.837
Sex	(female vs. male)	0.69	(0.35–1.41)	0.298			
Age	(<65 vs. ≥65 years)	1.07	(0.58–1.96)	0.825			
Smoking history	(never vs. ever)	0.64	(0.32–1.30)	0.208			
Clinical T stage	(T1–2 vs. T3–4)	1.06	(0.57–1.96)	0.842			
Clinical N stage	(N2 vs. N3)	0.64	(0.32–1.21)	0.166			
Contralateral mediastinal lymph node	(no vs. yes)	0.72	(0.38–1.32)	0.298			
SCF lymph node	(no vs. yes)	1.01	(0.54–1.86)	0.979			
FEV1 (%)	(≥70 vs. <70%)	0.80	(0.38–1.59)	0.529			
DLco (%)	(≥60 vs. <60%)	0.86	(0.38–1.84)	0.707			
PTV	(<550 vs. ≥550 cc)	1.61	(0.89–2.95)	0.121			
Fractional RT dose	(2.0 vs. 2.2 GyE)	1.15	(0.59–2.32)	0.691			
Total RT dose	(≤66 vs. >66 GyE)	0.76	(0.24–2.03)	0.605			
Both-lung D_mean_	(<20 vs. ≥20 GyE)	2.79	(1.51–5.19)	0.001	1.71	(0.62–4.74)	0.297
Both-lung V_5GyE_	(<65 vs. ≥65%)	1.86	(0.90–3.76)	0.087	0.61	(0.24–1.51)	0.292
Both-lung V_10GyE_	(<45 vs. ≥45%)	3.78	(2.04–7.10)	<0.001	4.37	(1.63–12.12)	0.004
Both-lung V_20GyE_	(<35 vs. ≥35%)	2.60	(1.40–4.86)	0.003	0.69	(0.20–2.23)	0.542

The foreparts of the parentheses were set as the reference group. Abbreviations: OR, odds ratio; CI, confidence interval; RT, radiation therapy; IMRT, intensity-modulated radiation therapy; PBSPT, pencil beam scanning proton therapy; SCF, supraclavicular fossa; FEV1, forced expiratory volume in 1 s; DLco, diffusing capacity of the lung for carbon monoxide; PTV, planning target volume; GyE, gray relative biologic effectiveness; BED10, biological effective dose with α/β of 10; V_XXGyE_ = volume receiving more than XX GyE.

**Table 5:** Patient, tumor, and treatment characteristics after propensity score matching and weighting.

Variables		Propensity Score Matching			IPTW		
		IMRT	PBSPT	SMD	IMRT	PBSPT	SMD
		*n* = 50	*n* = 25		*n* = 197.0	*n* = 53.6	
Sex	Male	42 (84.0)	21 (84.0)	<0.001	153.7 (78.7)	36.2 (67.5)	0.238
Age, years		67.7 (7.0)	67.5 (8.8)	0.033	62.0 (9.0)	60.1 (8.8)	0.217
ECOG	0–1	50 (100.0)	23 (92.0)	0.417	194 (98.5)	52.5 (97.9)	0.039
Histology	ADC	18 (36.0)	9 (36.0)	<0.001	110.0 (56.4)	35.3 (65.9)	0.196
	EGFR mutant	5 (10.00)	2 (8.0)	0.070	35.3 (17.9)	14.9 (27.8)	0.238
Clinical T stage	cT3–4	19 (38.0)	10 (40.0)	0.121	71.6 (36.4)	33.8 (63.2)	0.557
Clinical N stage	cN3	26 (52.0)	12 (48.0)	0.080	138.7 (70.4)	37.3 (69.6)	0.017
N3 region	Contralateral mediastinum	13 (26.0)	5 (20.0)	0.143	82.4 (41.8)	20.2 (37.7)	0.084
	Supraclavicular	14 (28.0)	9 (36.0)	0.172	68.7 (34.9)	24.1 (45.0)	0.207
Pre-treatment pulmonary function test							
FEV1, %		74.4 (20.0)	73.1 (24.2)	0.061	81.1 (18.7)	78.6 (21.7)	0.112
DLCO, %		71.5 (22.0)	67.0 (19.3)	0.216	78.0 (19.2)	82.3 (19.4)	0.121
Radiation therapy							
Gross tumor volume, cc		191.8 (188.8)	178.0 (151.2)	0.080	156.9 (162.8)	176.5 (133.6)	0.131
Clinical target volume, cc		443.4 (307.4)	453.6 (307.3)	0.033	378.9 (271.6)	440.3 (260.8)	0.231
Planning target volume, cc		704.8 (405.3)	734.4 (437.9)	0.070	628.0 (361.1)	711.7 (380.8)	0.226

Values are presented as number of patients (%) or mean (standard deviation). Abbreviations: IPTW, inverse probability of treatment weighting; IMRT, intensity-modulated radiation therapy; PBSPT, pencil beam scanning proton therapy; SMD, standardized mean difference; ADC, adenocarcinoma; EGFR, Epidermal growth factor receptor; FEV1, forced expiratory volume in 1 s; DLCO, diffusing capacity of the lung for carbon monoxide.

**Table 6:** Patient, tumor, and treatment characteristics after propensity score matching and weighting.

(Reference: IMRT)	PSM			IPTW		
	HR	(95% CI)	*p*-value	HR	(95% CI)	*p*-value
Locoregional control	0.46	(0.13–1.67)	0.236	0.87	(0.46–1.64)	0.668
Overall survival	1.69	(0.54–5.29)	0.371	1.39	(0.71–2.71)	0.339
	OR	(95% CI)	*p*-value	OR	(95% CI)	*p*-value
Acute esophagitis grade ≥ 3	2.91	(0.68–12.45)	0.151	5.33	(1.21–23.46)	0.028
Radiation pneumonitis grade ≥ 2	0.54	(0.34–1.04)	0.059	0.32	(0.09–1.15)	0.082

Abbreviations: IMRT, intensity modulated radiation therapy; PSM, propensity score matching; IPTW, inverse probability of treatment weighting; HR, hazards ratio; CI, confidence interval; OR, odds ratio.

## Data Availability

The datasets generated and analyzed during the current study are not publicly available due to institutional data protection law and confidentiality of patient data but are available from the corresponding author upon reasonable request in person.

## Supplementary Materials

The following are available online at https://www.mdpi.com/article/10.3390/cancers13143497/s1, Figure S1: Baseline pulmonary function tests according to treatment modality: forced expiratory volume in 1 s (A); diffusing capacity of the lung for carbon monoxide (B), Figure S2: Changes in pulmonary function test after treatment: forced expiratory volume in 1 s (A); diffusing capacity of the lung for carbon monoxide (B), Table S1: Dose volume parameters according to the treatment modality, Table S2: Comparison of target coverage and normal tissue sparing with matched intensity-modulated radiation therapy (IMRT) and intensity-modulated proton therapy (PBSPT) plans.
