# Supplementary material for: Clinical Outcomes of Pencil Beam Scanning Proton Therapy in Locally Advanced Non-Small Cell Lung Cancer: Propensity Score Analysis
Source: Cancers (Basel). 2021 Jul 13;13(14):3497. doi: 10.3390/cancers13143497 (PMC8307066; doi:10.3390/cancers13143497)
Supplement: Supplementary file 1 [file cancers-13-03497-s001.zip › cancers-1272360-supplementary.pdf]

cancers

MDPI

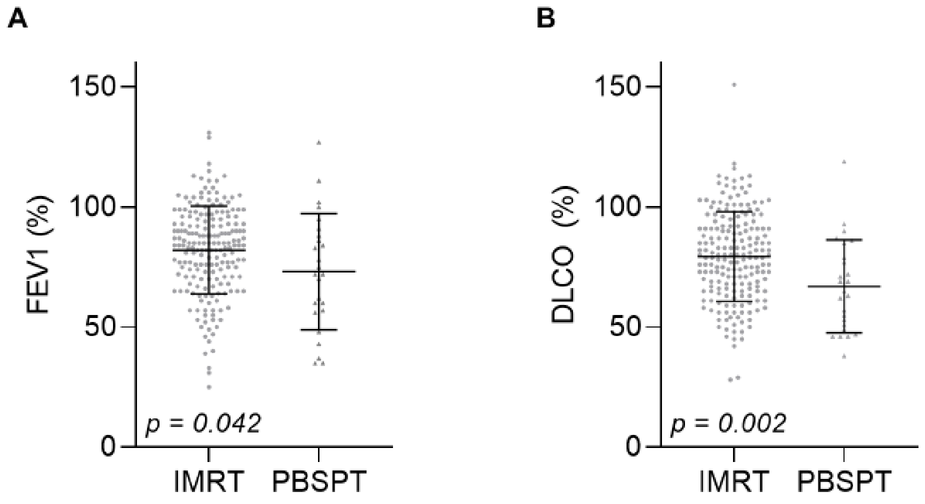

Figure S1. Baseline pulmonary function tests according to treatment modality: forced expiratory volume in 1 second (**A**); diffusing capacity of the lung for carbon monoxide (**B**). * Lines refer to median and interquartile range. Abbreviations: IMRT, intensity-modulated radiation therapy; PBSPT, pencil beam scanning proton therapy.

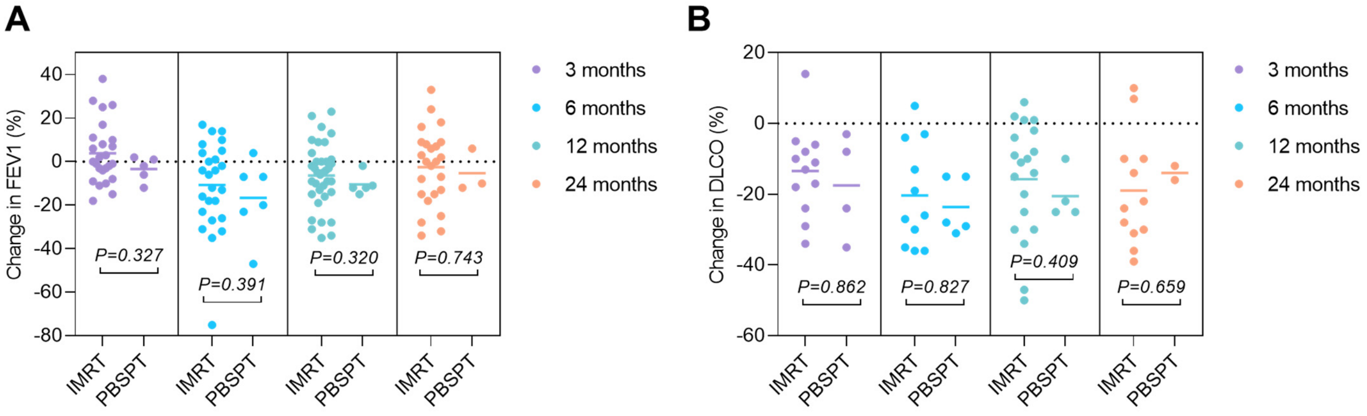

Figure S2. Changes in pulmonary function test after treatment: forced expiratory volume in 1 second (**A**); diffusing capacity of the lung for carbon monoxide (**B**). (Each dot represents results of pulmonary function tests from individual patients).

Table S1. Dose volume parameters according to the treatment modality.

		IMRT<br>*n* = 194	PBSPT<br>*n* = 25	*p*-value
Median total dose (range), GyE		66.0 (60.0-74.0)	66.0 (59.4-74.0)	0.580
Fractional dose, *n* (%)	2.0 GyE	9 (36.0)	9 (36.0)	0.398
	2.2 GyE	16 (64.0)	16 (64.0)	
Median biologically effective dose (range), Gy		80.5 (72.0-88.8)	80.5 (72.0-88.0)	0.650
		Median [IQR]	Median [IQR]	
Gross tumor volume, cc		98.8 [56.2;192.8]	118.4 [88.7;238.6]	0.154
CTV, cc		283.6 [184.8;475.9]	369.0 [263.5;512.6]	0.107
PTV, cc		532.6 [366.4;776.7]	611.4 [441.9;887.2]	0.150
$CTV_{95\%}$, %		99.9 [99.4;100.0]	99.9 [99.3;100.0]	0.905
$CTV_{100\%}$, %		96.2 [95.0;97.3]	96.7 [95.0;99.0]	0.314
$PTV_{95\%}$, %		97.1 [94.1;99.0]	94.8 [93.6;97.1]	0.013
Total lung				
Mean dose, GyE		18.9 [15.0;21.3]	14.0 [11.8;16.0]	<0.001
V_{5GyE}, %		54.7 [47.1;63.3]	32.9 [27.2;40.5]	<0.001
V_{10GyE}, %		43.3 [35.9;49.8]	28.6 [23.7;36.1]	<0.001

V_{20GyE}, %	32.1 [25.6;37.3]	23.9 [20.8;27.4]	<0.001
Esophagus			
Maximum dose, GyE	71.2 [68.7;72.9]	69.7 [68.6;71.5]	0.042
V_{45GyE}, %	27.5 [17.7;37.3]	26.2 [10.2;38.8]	0.901
V_{55GyE}, %	21.1 [10.1;29.5]	20.7 [6.3;32.2]	0.774
V_{66GyE}, %	7.0 [0.7;16.1]	9.5 [0.1;18.1]	0.484
Heart			
Mean dose, GyE	12.8 [6.8;20.9]	7.7 [5.3;12.6]	0.006
V_{30GyE}, %	14.6 [5.2;27.3]	9.1 [6.4;17.2]	0.091
V_{45GyE}, %	7.7 [2.8;17.3]	5.8 [3.0;11.2]	0.273
V_{50GyE}, %	5.7 [2.0;13.2]	4.8 [2.4;9.3]	0.517
Spinal cord			
Maximum dose, GyE	42.6 [40.5;44.6]	31.0 [22.6;38.4]	<0.001

Abbreviations: IMRT, intensity-modulated radiation therapy; PBSPT, pencil beam scanning proton therapy; SMD, standardized mean difference; GyE, gray equivalent; CTV, clinical target volume; PTV, planning target volume; $V_{XX\%}$ = volume receiving XX% of the prescription dose; V_{XXGyE} = volume receiving more than XX GyE.

Table S2. Comparison of target coverage and normal tissue sparing with matched intensity-modulated radiation therapy (IMRT) and intensity-modulated proton therapy (PBSPT) plans.

	**IMRT Median [IQR]**	**PBSPT Median [IQR]**	***p*-value**
Target			
CTV_{min}, GyE	55.8 [43.7;62.0]	67.0 [49.9;59.7]	0.711
CTV_{max}, GyE	73.3 [71.5;75.2]	72.7 [71.5;73.8]	0.442
$V_{95\%}$, %	99.8 [99.3;100.0]	99.9 [99.3;100.0]	0.173
$V_{100\%}$, %	97.4 [96.4;98.3]	96.7 [95.0;99.0]	0.275
PTV_{min}, GyE	35.0 [30.6;51.0]	39.8 [26.7;45.6]	0.916
PTV_{max}, GyE	73.2 [71.6;75.2]	72.7 [71.6;73.8]	0.252
$V_{95\%}$, %	96.7 [93.9;98.5]	94.8 [93.5;97.1]	0.075
$V_{100\%}$, %	85.3 [82.4;86.6]	85.8 [80.6;87.1]	0.653
Normal organ			
Both Lung			
D_{mean}, GyE	18.0 [15.1;23.1]	14.0 [11.8;16.0]	<0.001
V_{5GyE}, %	52.4 [48.7;69.6]	32.9 [27.2;40.5]	<0.001
V_{10GyE}, %	40.1 [34.8;49.8]	28.6 [23.7;36.1]	<0.001
V_{20GyE}, %	28.7 [26.9;38.5]	23.9 [20.8;27.4]	<0.001
Esophagus			
D_{max}, GyE	71.0 [65.2;73.1]	69.7 [68.6;71.5]	0.241
V_{45GyE}, %	22.4 [8.1;29.3]	26.1 [10.2;38.8]	0.017
V_{55GyE}, %	15.7 [4.1;25.9]	20.7 [6.3;32.2]	0.027
V_{66GyE}, %	4.3 [0;15.0]	9.5 [0.0;18.1]	0.001
Heart			
D_{mean}, GyE	14.1 [9.6;23.9]	7.7 [5.3;12.6]	<0.001
V_{30GyE}, %	16.0 [11.9;35.0]	9.1 [6.4;17.2]	<0.001
V_{45GyE}, %	8.3 [5.2;17.4]	5.8 [3.0;11.2]	0.000
V_{50GyE}, %	8.3 [3.7;13.7]	4.8 [2.4;9.3]	0.001
Spinal cord			
D_{max}, GyE	41.7 [39.3;44.0]	31.0 [22.6;38.4]	0.275

Abbreviations: IMRT, intensity-modulated radiation therapy; PBSPT, pencil beam scanning proton therapy; IQR, interquartile range; GyE, gray relative biologic effectiveness; $V_{XX\%}$ = volume receiving XX% of the prescription dose; D_{mean}, mean dose; D_{max}, maximum dose; V_{XXGyE} = volume receiving more than XX GyE.
